# Clinical manifestations and diagnostic methods in pulmonary angiosarcoma: protocol for a scoping review

**DOI:** 10.1186/s13643-017-0531-6

**Published:** 2017-07-10

**Authors:** Rachel Lim, Lea Harper, John Swiston

**Affiliations:** 1 Cumming School of Medicine, University of Calgary, Foothills Medical Centre, Room 933, North Tower, 1403-29 Street NW, Calgary, AB T2N 2T9 Canada; 2 University of British Columbia, 7th Floor, 2775 Laurel Street, Vancouver, BC V5Z 1M9 Canada; 3 Department of Medicine, 7th Floor, 2775 Laurel Street, Vancouver, BC V5Z 1M9 Canada

**Keywords:** Angiosarcoma, Neoplasm, Pulmonary, Scoping review

## Abstract

**Background:**

Angiosarcoma involving the lung can represent either primary or metastatic malignancy. Due to the rarity of this condition, knowledge surrounding the natural history and clinical presentation is scarce. The aim of this scoping review is to summarize the existing literature on pulmonary angiosarcoma, particularly as it pertains to the clinical presentation and ancillary tests used for diagnosis in addition to histopathology.

**Methods:**

We will conduct a systematic search using Ovid MEDLINE and EMBASE electronic databases. Two investigators will independently screen identified titles and abstracts to select articles reporting on pulmonary angiosarcoma. The data will be summarized in a narrative fashion and organized according to aspects of epidemiology, risk factors, clinical presentation, diagnostic methods, and treatment.

**Discussion:**

Scoping reviews are increasingly used to synthesize the evidence on a particular topic, to identify gaps in the literature, and to determine if future systematic reviews are feasible. In order to improve the care of patients with angiosarcoma, earlier recognition and diagnosis is required. This review will be valuable for highlighting the range of clinical presentations and the role of imaging and other diagnostic tools in the diagnosis of metastatic and primary pulmonary angiosarcoma.

**Systematic review registration:**

PROSPERO registration: CRD42017059052

**Electronic supplementary material:**

The online version of this article (doi:10.1186/s13643-017-0531-6) contains supplementary material, which is available to authorized users.

## Background

Angiosarcomas are rare, aggressive tumors arising from either vascular (hemangiosarcoma) or lymphatic (lymphangiosarcoma) endothelium. These account for less than two percent of all sarcomas and are further subdivided into cutaneous angiosarcoma, lymphoedema-associated angiosarcoma, radiation-induced angiosarcoma, primary breast angiosarcoma, and soft-tissue angiosarcoma [1, 2].

Cutaneous angiosarcoma has a median age of incidence of approximately 60 years, and there is a slight male predominance [3]. Cutaneous angiosarcoma has a predilection for head and neck location but can spread to any organ, most commonly the lung [1–3]. If angiosarcoma presents in the lung, it more likely represents metastatic disease rather than primary malignancy. Presentation varies widely including incidental findings on imaging, cough, constitutional symptoms, and hemoptysis [1].

Pneumothorax is a rare phenomenon, but there are reports of bilateral simultaneous pneumothoraces [3–6]. The association of angiosarcoma with pneumothorax is likely due to cystic lesions often found on imaging. Various mechanisms have been proposed for the development of cystic lesions including excavation of solid nodular lesions and infiltration of tumor cells into preexisting bullae or air sacs [7]. A mechanism described in 1973 postulated that necrotic nodules invade into a bronchus and pleural space leading to bronchopleural fistula formation [8]. In a literature review of previous case reports of pneumothorax from angiosarcoma, Chang et al. found that the scalp was the most common primary site [9]. An analysis of a Japanese autopsy registry observed that pneumothorax was limited to patients with angiosarcoma of the scalp [10].

In contrast, hemoptysis has been associated with both primary and metastatic angiosarcoma [3, 11–13]. Lung nodules are frequently present in these cases with pulmonary hemorrhage [3, 11–13]. There is no consensus on differentiating features in presentation or imaging between primary and metastatic pulmonary angiosarcoma. A review of 24 patients with metastatic angiosarcoma involving the lung found that the most common computed tomography (CT) signs were multiple solid nodules followed by multiple thin-walled cysts [9].

Other diagnostic methods have been reported with varying success. Fluorine-18 fluoro-2-deoxy-D-glucose positron emission tomography (18F-FDG PET) is commonly used in the evaluation of potentially malignant tumors but may contribute to diagnostic uncertainty in pulmonary angiosarcoma. While reports have demonstrated PET positivity in cardiac angiosarcoma [14], there are also reports of metastatic angiosarcoma lesions in the lung that were not metabolically active on PET-CT [1, 15].

Similarly, pleural fluid analysis has a variable yield in case reports [9, 10, 12, 13]. Ultimately, diagnosis relies on histopathology. The histological examination of angiosarcoma reveals abnormal, pleomorphic, malignant endothelial cells. Immunohistochemistry is important for confirming the diagnosis. Factor VIII, CD 31, CD34, Fli-1, Ulex europaeus agglutinin 1, and vimentin are typical endothelial markers [1]. The optimal method of tissue biopsy has not been studied but transbronchial biopsy (TBB), video-assisted thoracoscopic (VAT) pleural, and/or lung biopsy and surgical lung biopsy (SLB) have been tried [12, 16]. Tissue biopsy is invasive and is not always possible due to patient preferences or contraindications, particularly because angiosarcoma is often found in the elderly. Knowing which tests can aid in an earlier diagnosis may result in the prompt institution of treatment and may improve patient outcomes.

The prognosis of metastatic pulmonary angiosarcoma remains dismal, with 5-year survival rates of less than 40%. Most patients die within one year after diagnosis [3, 13]. Due to the rarity of this malignancy, the index of suspicion is low and diagnosis is often attained in advanced stages. The authors suspect that the current knowledge around the natural history and clinical presentation is largely based on case reports and case series, however, the landscape of evidence is unknown. The aim of this scoping review is to examine the existing literature on pulmonary angiosarcoma and to characterize the results as it pertains to the presentation and diagnosis of this rare disease.

## Methods

The methods of this scoping review will be based on the five stages outlined in the Arksey and O’Malley framework [17] as well as the guidelines from the Joanna Briggs Institute [18]. We will use the Preferred Reporting Items for Systematic Review and Meta-Analysis Protocols statement (see Additional file 1 for PRISMA-P checklist) for aspects applicable to a scoping review. There is a checklist being developed specifically for scoping reviews but is unavailable at this time. This protocol has been registered in the International Prospective Register of Systematic Reviews (PROSPERO). The study protocol registration number is CRD42017059052 and is available through PROSPERO: http://www.crd.york.ac.uk/PROSPERO.

### Stage 1: identifying research questions

Primary research question

I. What is known from the existing literature about the clinical manifestations in patients with primary or metastatic pulmonary angiosarcoma?

Other concepts to map existing literature, with supporting questions:

II. Risk factors for angiosarcoma (what is the frequency of reporting of known risk factors such as chemical exposure in the literature?)
III. Differentiation between primary and metastatic pulmonary angiosarcoma (do these differ in clinical presentation or imaging? What investigations should be pursued in the workup for metastatic disease?)
IV. Diagnostic methods (prior to pursuing biopsy, are there ancillary tests that can aid in the diagnosis of pulmonary angiosarcoma? What is the range of radiographic abnormalities? What method of biopsy should be recommended as first-line?)
V. Chemotherapy (as there are no guidelines, which patients are clinicians deciding to treat? Are they using single or combination chemotherapy?)

### Stage 2: identifying relevant studies

Two independent reviewers (RL and KS) will be performing the search in parallel. The online databases to be searched are Ovid MEDLINE (1946–present) and EMBASE (Excerpta Medica Database). The search of online databases will be restricted to humans and adults. The results will be downloaded into EndNote and duplicate results removed. The references of relevant full-length publications identified will be screened for additional studies relevant to the research question. Attempts will be made to contact authors to acquire full-length manuscripts for relevant abstracts if they are not readily available, or if information is missing from full-length publications.

Table 1 outlines the initial electronic search strategy. The first Boolean search will be done by using the term “or” to explode (search by subject heading) and map (search by keyword) the following MeSH (Medical Subject Headings) headings “hemangiosarcoma” or “vascular neoplasms” or “angiosarcoma”. The second Boolean search will be done using the terms “or” to explode and map “pulmonary” or “lung”. The two Boolean searches will be combined by using the Boolean term “and”. As suggested by the Joanna Briggs Institute [18], an iterative approach will be applied by collecting and analyzing the index terms, keywords, and title words from the studies included for full-text review during the initial search. If there are essential search terms missing from the initial search strategy then an additional search may be warranted using updated search terms.

**Table 1:** Details of electronic bibliographic database search strategies

Ovid MEDLINE	
1	Exp Hemangiosarcoma/
2	Hemangiosarcoma.tw
3	Exp Vascular neoplasms/
4	Angiosarcoma.mp
5	Angiosarcoma.tw
6	1 or 2 or 3 or 4 or 5
7	Pulmonary.mp
8	Pulmonary.tw
9	Exp Lung/
10	Lung.mp
11	6 or 7 or 8 or 9
12	6 and 11

### Stage 3: study selection

Two investigators (RL and LH) will independently screen citation titles and abstracts and review potentially relevant articles in full. We will consider any articles representing original research involving adults with pulmonary angiosarcoma. Included articles are required to report on the clinical presentation and diagnostic investigations pursued in a patient with biopsy-proven angiosarcoma involving the lung. The diagnosis of angiosarcoma will be based on either lung biopsy or extrapulmonary biopsy in conjunction with abnormal chest imaging. Our exclusion criterion will include non-English language publications, pediatric populations, animal studies, and narrative reviews. Studies which potentially replicate study participants will also be excluded.

If an agreement for abstract or full article inclusion cannot be reached between the two reviewers, an opinion will be requested from a third reviewer (JS). The degree of agreement between reviewers for each of these steps (full-text review, article inclusion) will be quantified with a kappa statistic with a 95% confidence interval.

### Stage 4: charting the data

Both reviewers will use the same template for data extraction; details are presented in Table 2. Data extraction will be done independently by each reviewer (LH, RL). The data extraction form will be piloted on the first ten studies to determine whether the information gathered is serving the purpose of the study.

**Table 2:** Description of data extracted from each included study

Type of data	
Study characteristics	Year of publication Authors Country of study Sample size Study design
Patient characteristics	Age Gender Ethnicity Comorbidities Smoking status History of prior chemical or occupational exposure History of prior radiation History of breast cancer or other malignancy
Disease characteristics	Duration from symptom onset to presentation Duration from symptom onset to diagnosis Respiratory symptoms at onset Extrapulmonary symptoms Physical examination findings
Results of Investigations	Chest radiograph Chest computed tomography Lung biopsy (TBB, VAT biopsy, SLB) Skin or other organ biopsies 18F-FDG PET Pleural fluid analysis Laboratory data
Treatment and survival outcomes	Chemotherapy regimens and duration Surgery Palliative care Patient outcomes (rehospitalization, death)

### Stage 5: collating, summarising, and reporting of results

The process of study inclusion in the scoping review will be tracked through a PRISMA flow diagram. A table mapping the distribution of studies according to the study design will be presented for both primary and metastatic disease.

The literature will be organized according to the aforementioned concepts; namely, clinical presentation, risk factors, epidemiology, diagnostic methods, and treatment. Data will be separated for primary versus metastatic pulmonary angiosarcoma. Frequencies and percentages will be calculated for categorical variables, and descriptive statistics, including means and standard deviation, for continuous variables. Analysis will be carried out in Stata version 14.0 (StataCorp, College Station, TX, USA).

## Discussion

Scoping reviews require similar expertise in finding and retrieving data as systematic reviews yet serve a different purpose altogether. In keeping with one of the roles proposed by Arksey and O’Malley [17], the main purpose of this scoping review is to explore the existing literature as it pertains to pulmonary angiosarcoma. Secondary to this, the authors will attempt to identify gaps in the literature and also determine if a systematic review is feasible for any diagnostic test with enough literature.

The key to improving outcomes for patients with pulmonary angiosarcoma is early recognition and accurate diagnosis, therefore, the priority of this scoping review is to focus on the clinical presentation. The lack of awareness of angiosarcoma often results in low clinical suspicion. This review will be valuable for outlining the typical presentations and discussing the roles played by imaging and other potential tools like 18F-FDG PET in the diagnosis of pulmonary angiosarcoma. Regarding other organs affected by angiosarcoma, readers can refer to a review by Gaballah et al. on the clinical and radiologic features of angiosarcoma affecting multiple organ systems including the breast, bone, spleen, and liver [19].

In rare conditions such as angiosarcoma, population-based studies or trials are unlikely to occur without significant effort. Systematic reviews of case reports and series of other uncommon conditions have proven their value by guiding clinical practice and future research [20–23]. We suspect that the majority of the literature will be case reports and series, however, we will not exclude other study designs as is typical for scoping reviews.

A limitation to scoping reviews is the lack of critical appraisal since the focus is on determining the sum and distribution of evidence. In addition, if the majority of evidence stems from case reports with their inherent biases, then it is not surprising that outcomes such as the frequency of clinical manifestations may be influenced by selective reporting of more unusual presentations such as bilateral pneumothoraces. The strength of this study will be the thorough, systematic review of all published literature on pulmonary angiosarcoma. The results of this scoping review will be valuable for informing clinicians and researchers in the development of future guidelines for the diagnosis and management of pulmonary angiosarcoma, as well as priorities for future research.

## Additional file

Additional file 1: PRISMA-P file, PRISMA-P 2015 Checklist. (DOCX 38 kb)

## Abbreviations

18F-FDG PET: Fluorine-18 fluoro-2-deoxy-D-glucose positron emission tomography
CT: Computed tomography
EMBASE: Excerpta Medica Database
MEDLINE: Medical Literature Analysis and Retrieval System Online
MeSH: Medical Subject Headings
PRISMA-P: Preferred Reporting Items for Systematic review and Meta-Analysis Protocols
PROSPERO: International Prospective Register of Systematic Reviews
SLB: Surgical lung biopsy
TBB: Transbronchial biopsy
VAT: Video-assisted thoracoscopic
